# Mining and evolution analysis of lateral organ boundaries domain (*LBD*) genes in Chinese white pear (*Pyrus bretschneideri*)

**DOI:** 10.1186/s12864-020-06999-9

**Published:** 2020-09-21

**Authors:** Bobo Song, Zikai Tang, Xiaolong Li, Jiaming Li, Mingyue Zhang, Kejiao Zhao, Hainan Liu, Shaoling Zhang, Jun Wu

**Affiliations:** 1 Center of Pear Engineering Technology Research, State Key Laboratory of Crop Genetics and Germplasm Enhancement, Nanjing Agricultural University, Nanjing, 210095 China; 2 Shenzhen Branch, Guangdong Laboratory for Lingnan Modern Agriculture, Genome Analysis Laboratory of the Ministry of Agriculture, Agricultural Genomics Institute at Shenzhen, Chinese Academy of Agricultural Sciences, Shenzhen, China

**Keywords:** *LBD* gene family, *Pyrus*, Synteny analysis, Gene expression, Anthocyanins biosynthesis

## Abstract

**Background:**

The lateral organ boundaries domain (*LBD*) gene is a plant-specific transcription factor that plays a critical role in diverse biological processes. However, the evolution and functional divergence of the *LBD* gene family has not yet been characterized for the Chinese White Pear.

**Results:**

In our study, a total of 60 *PbrLBDs* were identified in the pear genome. The *PbrLBD* gene family was divided into two classes based on gene structure and phylogenetic analysis: class I (53) and class II (7). *Cis*-acting element analysis results suggested that *PbrLBDs* may participate in various biological processes, such as flavonoid biosynthetic and stress response. Synteny analysis results indicated that segmental duplication played a key role in the expansion of the *PbrLBD* gene family. The mean Ks and 4DTv values showed that the *PbrLBD* gene family had undergone only one recent whole-genome duplication event occurring at 30–45 MYA. Purifying selection was a primary force during the *PbrLBD* gene family evolution process. Transcriptome data analysis revealed that 10 *PbrLBDs* were expressed in all six examined tissues, and 73.33% of members in the *PbrLBD* gene family were expressed in pear sepal. qRT-PCR was conducted to verify the expression levels of 11 *PbrLBDs* in these six tissues. Specifically, *PbrLBD20*, *PbrLBD35* and *PbrLBD53* genes were down-regulated when anthocyanin concentrations were high, whereas *PbrLBD33* was significantly up-regulated in pear when anthocyanin concentrations were high. Furthermore, *PbrLBD20*, one of the candidate genes related to anthocyanins was localized in the nucleus.

**Conclusions:**

Our analysis provides valuable information for understanding the evolution of the *PbrLBD* gene family, and provides new insights into the regulation of pear pigment metabolism and lays a foundation for the future disclosure of the molecular mechanism of *LBD* gene regulating flavonoid metabolism.

## Background

Lateral organ boundaries domain (*LBD*) genes are plant-specific transcription factors (TFs) that play important roles in the growth and development of plants. They contribute to abiotic stress responses, anthocyanin biosynthesis, nitrogen metabolism and development of lateral organ, among other processes [1–4]. According to the structural characteristics of LBD, the *LBD* genes were divided into two subclasses, class I and class II [5, 6]. The class I genes encode a conserved LOB domain composed of a conserved CX2CX6CX3C zinc finger-like domain at the N terminal and a conserved LX6LX3LX6L leucine zipper-like domain [5, 7]. However, the class II genes only contain a conserved CX2CX6CX3C zinc finger-like coiled-coil domain [8]. A previous study showed that the two class I domains are involved in different roles. The CX2CX6CX3C zinc finger-like domain was associated with DNA binding, while the LX6LX3LX6L leucine zipper-like domain was used for protein-protein interactions [5].

The function of many *LBD* genes has been previously identified in several model species. For example, *AS2*, which is related to the formation of flat symmetric leaves, is one of the most extensively investigated members of the *LBD* gene family. In *Arabidopsis thaliana*, it was shown that *Knotted1-like homeobox* (*KNOX*) genes function in maintaining an active meristem. However, the *AS1*-*AS2* complex was shown to promote meristem cell differentiation by repressing expression of *KNOX* genes. One previous study showed that *ARF3* (*ETTIN/AUXIN RESPONSE FACTOR3*) was an important determinant for specification of adaxial cells in leaves, while the expression of *ARF3* was directly repressed by *AS2* and *AS1* [9]. This result indicated that *AS2* and *AS1* indirectly control the formation of adaxial cells in leaves. In addition, the development of plant roots also was regulated by several *LBD* genes. As a member of the maize *LBD* gene family, *ra2* can directly regulate the development of stem cells in maize branch meristems. The *ramosa2* (*ra2*) mutant causes an increase in the number of branches and long branches to replace short branches compared with normal maize [10]. It has been previously shown that *AtARF7* and *AtARF19*, as early auxin response factors, regulate the formation of the lateral root in *Arabidopsis* [3]. Recent studies further revealed that *AtARF7* and *AtARF19* directly regulate the expression of *AtLBD16* and *AtLBD29*, which induce formation of the lateral root. Moreover, *AtLBD18* is also regulated by *AtARF7* and *AtARF19*, and functions in lateral root formation. In rice, *ARL1* encodes a predicted protein containing a LOB domain with a broad range of expression in various tissues, including lateral and adventitious root primordia, tiller primordia, vascular tissues, scutellum and young pedicels. It also plays an important role in the development of adventitious roots. Meanwhile, several *LBD* genes also are involved in nitrate metabolism and anthocyanin synthesis. For example, one study showed that *ASL39/LBD37*, *ASL40/LBD38* and *ASL41/LBD39* were induced by N/NO_3_^−^, and negatively regulated anthocyanin biosynthesis in *Arabidopsis thaliana* [2].

To date, the *LBD* gene family has been systematically investigated at the whole-genome level for some plants, such as *Arabidopsis thaliana*, maize (*Zea mays*), rice (*Oryza sativa*), poplar (*Populus trichocarpa*), apple (*Malus domestica*), grape (*Vitis vinifera*) and tomato (*Solanum lycopersicum*) [5, 11–14]. Pear, one of the most important *Rosaceae* fruit trees, is widely cultivated all over the world. However, till now there has been insufficient information on *LBD* genes in pear, making functional characterization of *PbrLBD* genes difficult. The release of the Chinese White Pear genome (*Pyrus bretschneideri*) has provided an unprecedented opportunity to identify its *LBD* gene family at the whole-genome level. In the present study, we performed comprehensive analysis on genome-wide identification, chromosome distribution, genomic structure, and evolutionary and expression patterns of *PbrLBD* genes, which will establish a solid foundation for functional characterization of *PbrLBD* genes in the future.

## Results

### Whole-genome characterization of *PbrLBD* genes in Chinese white pear

In the present study, two strategies were independently performed to identify the candidate *LBD* gene set in the pear genome. First, a Hidden Markov Model search (HMMsearch) was performed against whole-genome protein sequences of pear using the HMM profile (PF03195) of the LOB domain. Second, a local BLASTP analysis was performed to scan the whole-genome protein sequences of pear using the *Arabidopsis thaliana* LBD proteins as query sequences [15]. Initially, we retained the common protein sequences identified by both methods. To further verify the reliability of candidate *LBD* genes, the SMART Tool, CDD tool and InterProScan tool were used to detect the completeness of the LOB domain of candidate proteins. As a result, four candidate genes with incomplete LOB domain were removed. Based on the location of candidate *LBD* genes, we also removed five genes that were anchored on scaffolds. Consequently, a total of 60 non-redundant and complete *LBD* genes were identified in the pear genome for further analysis. According to the presence/absence of the LX6LX3LX6L leucine zipper-like domain of LBD proteins, 60 *PbrLBD* genes were divided into two groups: 53 members belonged to class I and 7 members belonged to class II (Additional file 1: Figure S1). To distinguish members of the *PbrLBD* gene family, we named each of *PbrLBD* gene based on their order on the chromosomes (Additional file 2: Table S1), namely, *PbrLBD1*-*PbrLBD60*. In addition, we further analyzed the physical and chemical properties of 60 LBD proteins. The ExPASy Proteomics Server, an online proteomics and sequence analysis tool, was used to predict the molecular weights and isoelectric points of *PbrLBD* protein sequences. The length of *PbrLBD*-encoded protein sequences ranged from 150 (*PbrLBD52*) to 475 (*PbrLBD7*) amino acids. Protein masses ranged from 168.23 kD (*PbrLBD52*) to 50.74 kD (*PbrLBD7*), and protein pIs ranged from 4.56 (*PbrLBD4*) to 9.22 (*PbrLBD13*) (Additional file 2: Table S1).

#### Chromosome location of *PbrLBD* genes in the pear genome

To further investigate the distribution pattern of *PbrLBD* genes on chromosomes, we mapped all the members of the *PbrLBD* gene family in the pear genome. Mapchart software was used to show the location distribution of *PbrLBD* genes across the pear genome (Additional file 3: Figure S2). As a result, we found that 60 *PbrLBD* genes were unevenly distributed in the pear genome, with the exception of chromosome 4, chromosome 13 and chromosome 16. Chromosome 5 contained the largest number of *PbrLBD* genes (8 members), followed by chromosome 1 and chromosome 15, which each contained 7 *PbrLBD* genes. However, chromosome 6, chromosome 8 and chromosome 12 only contained one *PbrLBD* gene each. Three or more *PbrLBD* genes were mapped on chromosomes 2, 3, 7, 9, 10, 11, 14 and 17. Interestingly, we found that most *PbrLBD* genes existed in the form of gene clusters, suggesting that the *PbrLBD* family had undergone a WGD event/segmental duplication or tandem duplication.

### Phylogenetic analysis of the *PbrLBD* genes

**Fig. 1:**
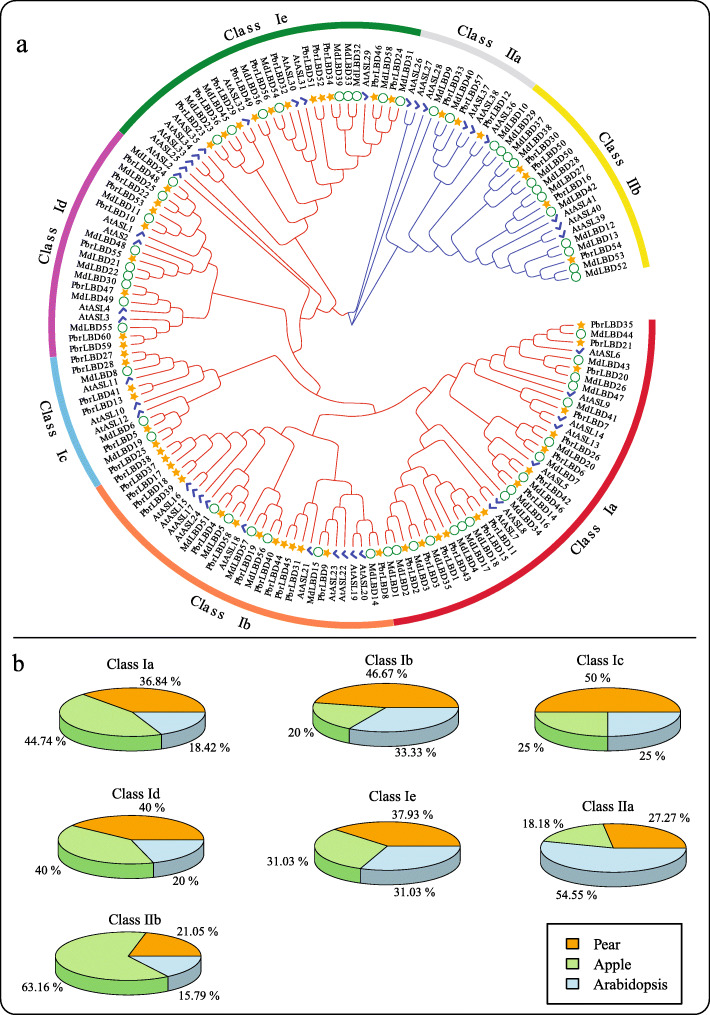
Phylogenetic tree and the distribution of *LBD* genes of three species in seven subgroups, including class Ia-Id, class IIa and class IIb. (**a**) A phylogenetic tree of LBD proteins of pear, apple and *Arabidopsis*. A phylogenetic tree of LBD protein family was constructed by Mega-X using the Neighbor–Joining (NJ) method and 1000 bootstraps. The red branches indicates the class I genes, and the blue branches indicates the class II. The yellow star, green circle and blue tick indicates the LBD protein in pear, apple and *Arabidopsis*, respectively. (**b**) The percentage of *LBD* genes of three species in each subgroup. The orange part indicates the proportion of *LBD* genes in pear; The green part indicates the proportion of *LBD* genes in apple; The blue part indicates the proportion of *LBD* genes in *Arabidopsis*

The phylogenetic tree is widely used to show the evolutionary relationship of gene families. A neighbour-joining (NJ) phylogenetic tree was constructed with the Mega-X program using the full-length LBD proteins of pear, apple and *Arabidopsis* (Fig. 1a). According to the phylogenetic tree results, a total of 168 LBD proteins from three species were phylogenetically categorized into seven subgroups, namely, class Ia, Ib, Ic, Id, Ie, and class IIa and IIb. In apple, 17, 6, 3, 8, 9, 2 and 12 *MdLBD* genes fell within the subgroups, respectively. In *Arabidopsis*, 7, 10, 3, 4, 9, 6 and 3 *AtASL* genes were clustered into the seven subgroups, respectively. In pear, class Ia and class Ib included the highest number of *PbrLBD* genes (14), followed by class Ie, which contained 11 *PbrLBD* genes (Fig. 1b). This result provides further evidence that class I contains a greater percentage of *LBD* genes than class II. It is important to note that most sister pairs of subclades consisted of *PbrLBDs* and *MdLBDs* (such as, *PbrLBD35*/*MdLBD44*, *PbrLBD20*/*MdLBD26*, *PbrLBD14*/*MdLBD16*, etc.), while most of the *AtLBD* genes were separate from *PbrLBD* genes and *MdLBD* genes (such as, *ASL2*, *ASL25*, *ASL26* and *ASL27*). This result suggests that the *PbrLBD* genes and *MdLBD* genes had a closer evolutionary relationship, and consistent with the fact that apple and pear evolved from the same ancestor.

### Conserved motif analysis and gene structural analysis of *PbrLBD* genes

**Fig. 2:**
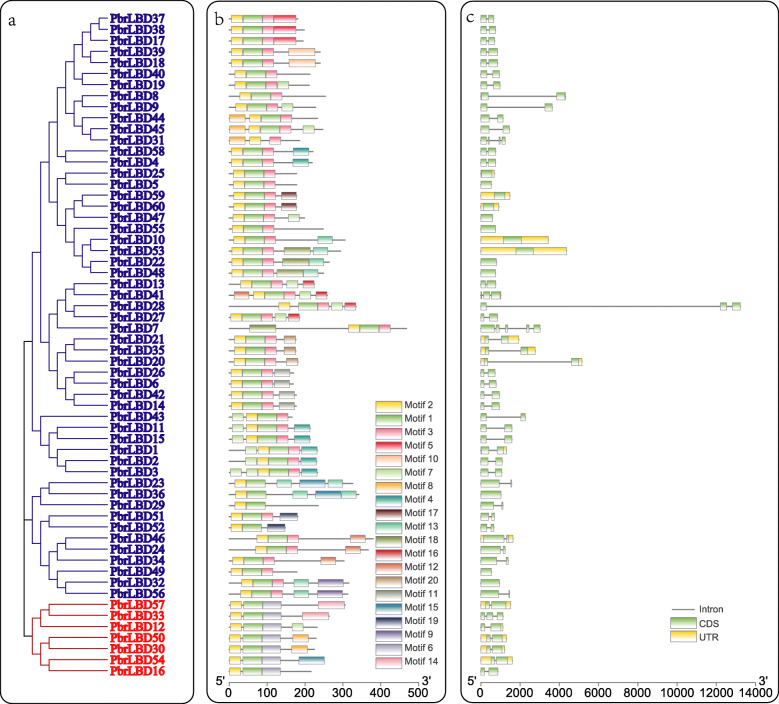
The phylogenetic relationship, conserved motifs and gene structure of PbrLBD proteins. (**a**) A phylogenetic tree of PbrLBD protein family was constructed by Mega-X using the Neighbor–Joining (NJ) method and 1000 bootstraps. The blue branches indicate class I; the red branches indicate class II. (**b**) The distribution of conserved motifs across 60 PbrLBD protein. A total of 20 motifs were predicted using MEME (Multiple Em for Motif Elicitation) tool in our study. The scale bar indicates 100 aa. (**c**) The gene structure of *PbrLBD* genes, including intron, exon, UTR. The black line indicates intron; the green rectangle indicates exon; the yellow rectangle UTR. The scale bar indicates 2 kb

To identify gene structures and evolutionary trajectories of *LBD* genes in pear, we investigated exon-intron compositions of the 60 *PbrLBDs*. The number of introns of the *PbrLBD* genes ranged from zero to four, suggesting that the introns of some *PbrLBDs* were lost during evolution. The *PbrLBD7* gene contained five exons and four introns. In addition, the genes clustered into subclades had similar gene structures (Fig. 2a, c). Most members of class II had two exons and two UTR regions, whereas no UTR regions were detected for most members of class I.

The MEME (Multiple Em for Motif Elicitation) tool was used to predict the conserved motifs of PbrLBD proteins (Fig. 2b) [16]. A total of 20 conserved motifs were identified in our study, named motif 1–20. As a result, the numbers and types of conserved motifs in PbrLBD protein sequences were relatively conserved. Most members clustered into the same subclade had similar motif structures, suggesting that proteins from the same subclade may have similar functions. Motif 1 and motif 2 were basic regions of the LOB domain that were detected in all members of the *PbrLBD* gene family. Class I members had motif 1 (CX2CX6CX3C), motif 2 (GAS blocks) and motif 3 (LX6LX3LX6L), while Class II members lacked motif 3 (LX6LX3LX6L). This result provided further evidence to support division of the *PbrLBD* gene family into two clusters.

### Cis-acting elements analysis in the putative promoter of *PbrLBD* genes

**Fig. 3:**
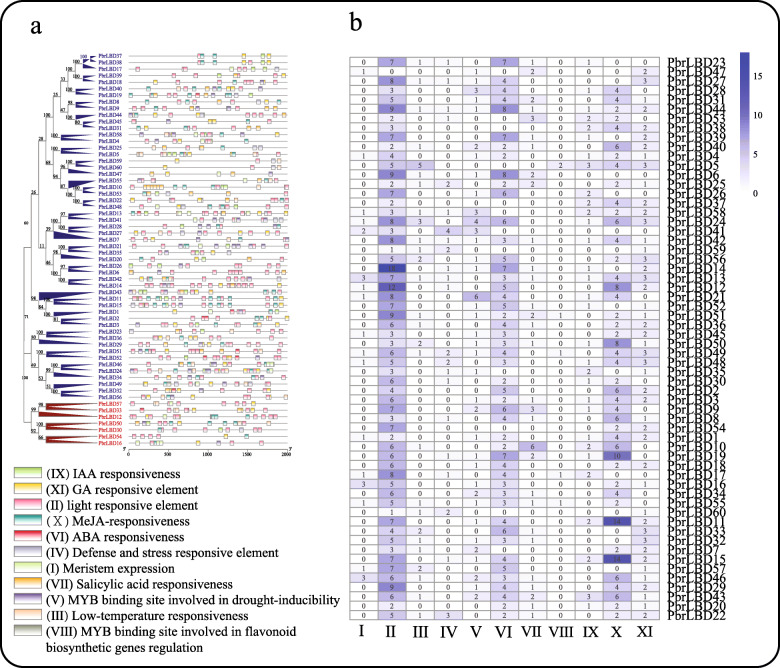
The cis-acting elements on putative promoters of *PbrLBD* genes. (**a**) The distribution of cis-acting elements on putative promoters of *PbrLBD* genes. A phylogenetic tree of *PbrLBD* protein family was constructed by Mega-X using the Neighbor–Joining (NJ) method and 1000 bootstraps. The blue branches of phylogenetic tree indicate *LBD* genes in class I; the red branches of phylogenetic tree indicate *LBD* genes in class II. (**b**) The number of cis-acting elements on putative promoters of *PbrLBD* genes. A total of eleven cis-acting elements were investigated in our study, including: (I) Meristem expression, (II) light responsive element, (III) Low-temperature responsiveness, (VI) ABA responsiveness, (V) MYB binding site involved in drought-inducibility, (VI) ABA responsiveness, (VII) Salicylic acid responsiveness, (VII) MYB binding site involved in flavonoid biosynthetic genes regulation, (IX) IAA responsiveness, (X) MeJA-responsiveness, (XI) GA responsive element

In general, the gene function was influenced by the distribution of cis-acting elements in the promoter region [17]. In this study, the region 2000 bp upstream (putative promoter sequences) of the transcription initiation site of *PbrLBD* genes was defined as the putative promoter region. The putative promoter sequences of *PbrLBD* genes were then submitted to the PlantCARE database to search for cis-acting elements (Fig. 3) [18]. As a result, a total of 266 cis-acting elements for plant growth and development were identified, and we selected 11 important cis-acting elements for further analysis (Fig. 3). In Fig. 3a, a diversity of distribution patterns of cis-acting elements was observed in the promoter region of *PbrLBD* genes, suggesting that the expression of *PbrLBD* genes may be regulated by various factors, such as, light, abscisic acid (ABA), methyl jasmonate (MeJA), gibberellin (GA) response element and myeloblastosis (MYB) binding site involved in drought-inducibility. For hormone-related elements, the ABA-responsive and MeJA-responsive elements were most frequently found in the pear LBD gene promotors. Previous studies had reported that four auxin-inducible *LBD* genes (*LBD16/ASL18*, *LBD17/ASL15*, *LBD18/ASL20* and *LBD29/ASL16*) were regulated by *AUXIN RESPONSE FACTOR7* (*ARF7*) and *ARF19* to control the formation of lateral root [3, 7]. In our study, the IAA responsiveness element (*IX*) were predicated on the putative promotor of 32 *PbrLBD* genes, this result indicated that the functions of *PbrLBD* genes might be regulated by auxin. It is notable that the element related to MYB binding site involved in flavonoid biosynthetic gene regulation was predicted in five *PbrLBD* genes (*PbrLBD5, PbrLBD17, PbrLBD49, PbrLBD51 and PbrLBD55*), suggesting that they may play important functions in the process of flavonoid biosynthesis.

### Synteny analysis reveal the expansion of the *LBD* gene family

**Fig. 4:**
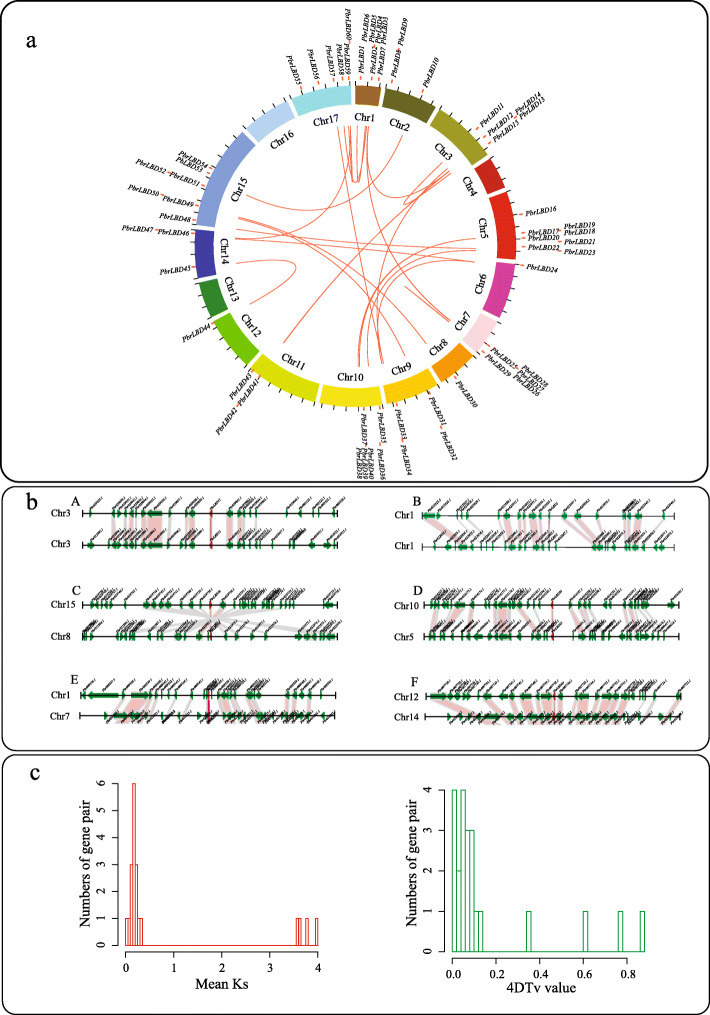
The evolution analysis of *PbrLBD* gene family. (**a**) Distribution and synteny of *PbrLBD* gene family in pear genome. The circle picture was plotted by the Circos software (version 0.69.2). The red points mark the position distribution of *LBD* gene on 17 pear chromosomes. The red lines show the collinear gene pairs of *LBD* gene family. (**b**) Segmental duplication between members of *PbrLBD* family. (A) *PbrLBD11* (*Pbr029040.1*) and *PbrLBD15* (*Pbr033910.1*), (B) *PbrLBD1* (*Pbr033910.1*) and *PbrLBD2* (*Pbr022066.1*), (C) *PbrLBD50* (*Pbr019737.1*) and *PbrLBD30* (*Pbr021980.1*), (D) *PbrLBD36* (*Pbr016186.1*) and *PbrLBD23* (*Pbr000434.1*), (E) *PbrLBD5* (*Pbr009548.1*) and *PbrLBD25* (*Pbr010928.1*), (F) *PbrLBD44* (*Pbr007737.1*) and *PbrLBD45* (*Pbr016876.1*). The figure shows a region of 100 kb on each side flanking the *PbrLBD* genes. Duplicated gene pairs are connected with bands. Chromosome segments are indicated by black horizontal lines, and a broad line with arrowhead represents a gene and its transcriptional orientation. Text indicates the gene ID. The *PbrLBD* genes are shown in red, duplicated genes and other genes are shown in green; and homologous genes are linked by the red line. (**c**) The distribution of mean Ks and 4DTv value of 22 *PbrLBD* duplicated gene pairs. In the left figure, the x-axis shows the mean Ks value, and the y-axis shows the number of gene pairs. In the right, the x-axis shows the 4DTv value, and the y-axis shows the number of gene pairs. The data were plotted using the hist function in R

Previous studies have reported that the expansion of gene families is driven by different gene duplication modes, including whole-genome duplication (WGD)/segmental duplication, dispersed duplications (DD), tandem duplication (TD), proximal duplication (PD), and transposed duplication (TRD). To explore which duplication types drove the expansion of the *PbrLBD* gene family, a synteny analysis was conducted using the MCScanX software package (Fig. 4). Then, the DupGen_finder tool was used to dissect gene duplication types with a priority of duplicate genes as follows: WGD > TD > PD > TRD > DD. As a result, 58 *PbrLBD* genes were assigned to one of five different duplication types. Consequently, 76.67% (44) genes in the *PbrLBD* gene family were duplicated and retained from WGD/segmental duplication types, followed by DD (6, 10%), TD (6, 10%), PD (1,1.67%) and TRD (1, 1.67%) (Additional file 4: Figure S3). This result shows that lineage-specific WGD/segmental duplication events were the major force in the expansion of the *PbrLBD* gene family.

To further investigate the evolutionary mechanisms of the *PbrLBD* gene family, we performed a method described on the Plant Genome Duplication Database (PGDD) to identify the synteny blocks in the pear genome. A total of 24 synteny blocks containing *PbrLBD* genes were identified. The 22 duplicated gene pairs from the *PbrLBD* gene family were mapped in the 19 synteny blocks, named synteny block 1–19 (Additional file 5: Table S2 and Fig. 4a). Among these synteny blocks, we found that several were strongly conserved. For example, synteny block 4 contained over 140 syntenic gene pairs. In order to further verify the reliability of synteny analysis, we investigated the distribution of the genes and the duplicated gene pairs in the 100-kb upstream and downstream regions of *PbrLBD* genes (Additional file 6: Table S3 and Fig. 4b). We found that most *PbrLBD* genes were located in 200-kb conserved synteny blocks. These results provided further evidence that expansion of the *PbrLBD* gene family was mainly driven by a WGD/segmental duplication event.

### Estimating dates and driving forces for evolution of the *PbrLBD* gene family

The number of synonymous substitutions per site (Ks) is extensively used to calculate the approximate occurrence dates of WGD/segmental duplication events. Our previous studies indicated that two WGD events had occurred during pear genome evolution, including an ancient WGD event (Ks ~ 1.5–1.8, approximately 140 MYA) derived from a paleohexaploidization (γ) event and a relatively recent WGD event (Ks ~ 0.15–0.3, approximately 30–45 MYA) [19, 20]. To further investigate the evolution dates *PbrLBD* gene duplication, mean Ks values of *PbrLBD* duplicated gene pairs were estimated to trace the date of WGD/segmental duplication events. Table S3 shows the mean Ks values for 22 *PbrLBD* paralogs in the syntenic region. The values ranged from 0.01 to 3.99. The gene sequences of several paralogs were completely consistent, which indicated that no mutations occurred between these gene pairs (*PbrBD27*/*PbrBD28*, *PbrBD11*/*PbrBD15*). Most of the mean Ks values of the 22 paralogs fell in the range from 0.15 to 0.30 (Fig. 4), suggesting that the duplication of these gene pairs may have taken place during the relatively recent WGD event, approximately 30–45 MYA. However, the mean Ks value of *PbrLBD* paralogs were not mapped to the region with values from 1.5 to 1.8, suggesting that the *PbrLBD* gene family had not undergone the ancient WGD event (~ 140 MYA). Surprisingly, three paralogs (*PbrBD18*/*PbrBD40*, *PbrBD33*/*PbrBD12* and*PbrBD34*/*PbrBD24*) exhibited relatively higher mean Ks values (3.61–3.98), suggesting that they duplicated during a more ancient duplication event.

Recently, Wu et al. reported that the pear genome underwent two significant groups of synteny blocks: a recent whole-genome duplication event (4DTv ([4-fold synonymous (degenerative) third-codon transversion]) ~ 0.08) and an ancient WGD event (4DTv ~ 0.5) [20]. To test these suppositions, we calculated the 4DTv values of 22 paralogs using our in-house scripts. The distribution of 22 paralogs ranged from 0 to 0.86 (Fig. 4c), and most members exhibited a strong peak with a 4DTv-value at approximately 0.08. This result further supported the conclusions of our mean Ks value *analysis*, which indicated the *PbrLBD* gene family had undergone only one WGD event occurring 30–45 MYA.

Three DNA mutation types have been identified in genome evolution, including beneficial, neutral and harmful mutations [21]. In nature, most neutral and harmful mutations are eliminated through purifying selection, while beneficial mutations are retained during the evolution process. The accumulation of beneficial mutations is important for plants and animals to respond to a complex and changing environmental conditions [22]. To identify which selection pressures were driven by the evolution of *PbrLBD* genes, the ka/ks ratios of 22 *PbrLBD* paralogs were calculated using the Kaks_calculator tool. A ka/ks ratio equal to one indicates neutral selection, less than one indicates negative selection (purifying selection) and higher than one indicates positive selection (Darwinian selection). Results showed that all ka/ks ratios of *PbrLBD* paralogs were less than 1, suggesting that negative selection (purifying selection) was the primary driving force for evolution of the *PbrLBD* gene family.

#### Expression analysis of *PbrLBD* genes by EST database

Expression sequence tags (EST) is the part of cDNA sequence from different tissues, the length ranged from 300 to 1000 bp. One EST represents an expression gene at one time in one cell or tissues. Expression sequence tags (EST) can provide further evidence to support the accuracy of the members of gene family from transcription level [23, 24]. To verify the accuracy of *PbrLBD* gene identification, a BLASTN search was performed to scan the ‘Dangshansuli’ pear expressed sequence tag (EST) database using all *PbrLBD* gene sequences as the query. A total of 54 EST hits were found for the *PbrLBD* gene family, with hits occurring for 33 of the genes (E-value<10e-10, identity> 95%, length > 200 bp). Three genes (*PbrLBD21*, *PbrLBD35* and *PbrLBD24*) had the greatest numbers of EST hits, with four EST hits each. This result provided further evidence that the *LBD* gene set is reliable. However, there were 27 *PbrLBD* genes that did not hit any tags from the Chinese White pear EST database. This may be due to there has been little research on *LBD* genes in pear.

### Expression patterns of *PbrLBDs* in different tissues of pear

**Fig. 5:**
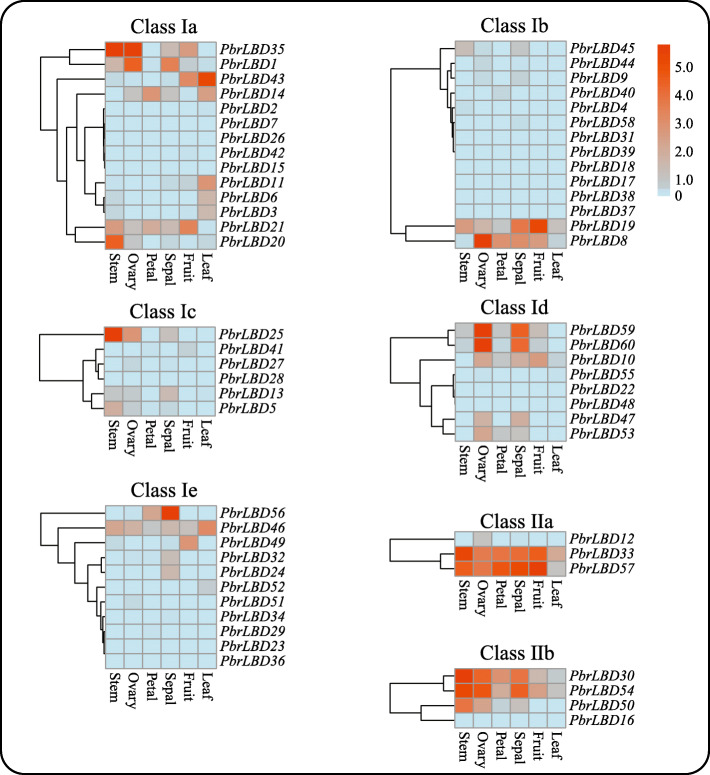
Heatmap of expression level of 60 *PbrLBD* genes at six different tissues, including stem, ovary, petal, sepal, fruit and leaf. Based on the result of phylogenetic tree (Fig. 1a), the *LBD* gene family was divided into seven subgroups: class Ia, class Ib, class Ic, class Id, class IIa, class IIb. The heatmaps were plotted by heatmap.2, a function of the gplots package in R. Red color indicates high expression and blue indicates low expression. The color scale at the top right indicates RPKM (reads per kilobase per million) values normalized by log2

Transcriptome sequencing analysis was conducted using transcriptome data from six different tissues of ‘Dangshansuli’ cultivar including stem, ovary, petal, sepal, fruit and leaf [25]. The RPKM (reads per kilobase per million) values were measured to represent the expression level of *PbrLBD* genes, and a value of RPKM equaling 0 represented that the gene had no expression in libraries. The expression patterns of 60 *PbrLBD* genes were shown in Fig. 5 using heatmap.2, a function of the gplots package in R. Among 60 *PbrLBD* genes, 11 *PbrLBD* genes were expressed in all six different tissues, indicating that they have various roles in the development of different tissues. 51.67, 60.00, 48.33, 73.33, 40.00 and 35.00% of all *PbrLBDs* were expressed in six different tissues corresponding to stem, ovary, petal, sepal, fruit and leaf, respectively. Thirty-nine genes were expressed in at least one of six different tissues, although the transcript abundance of several genes was relatively lower for certain tissues. However, 10 *PbrLBD* genes were not expressed in any of the six different tissues, suggesting that a portion of *PbrLBDs* were functional redundancies. As shown in Fig. 1b, the genes in class Ib (46.67%) and Ic (50%) have substantial expansion in the pear genome. According to transcriptome data, we found that seven members in class Ib were not expressed in anyone tissues, suggesting that these *PbrLBDs* in class Ib were functional redundancies. Among class Ic, three *LBD* transcription factors *PbrLBD25*, *PbrLBD13* and *PbrLBD5* exhibited relatively high expression level in stem, suggesting that these three genes might occur neofunctionalisation. In summary, the result of transcriptome sequencing analysis supported that *PbrLBD* genes played important roles in multiple biological processes.

**Fig. 6:**
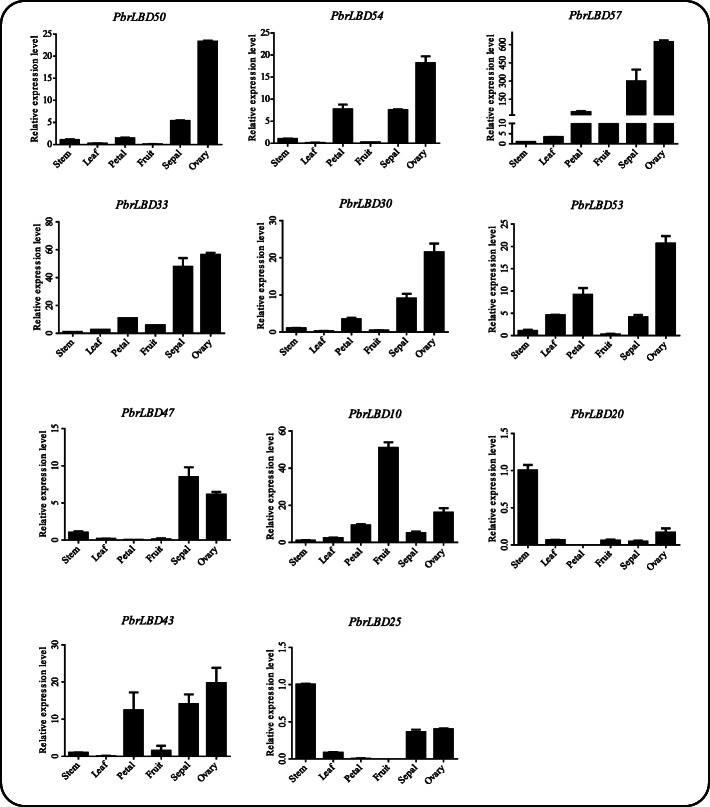
Expression patterns of eleven *PbrLBD* genes at six different tissues, including *PbrLBD50*, *PbrLBD54*, *PbrLBD57*, *PbrLBD33*, *PbrLBD30*, *PbrLBD53*, *PbrLBD47*, *PbrLBD10*, *PbrLBD20*, *PbrLBD43*, *PbrLBD25*. Relative expression levels of *PbrLBD* genes were detected by qRT-PCR experiment. The x-axes represent six different tissues, including stem, ovary, petal, sepal, fruit and leaf; the y-axes represent relative expression level of *PbrLBD* gene. Error bars indicate three technical replicates derived from one bulked biological replicate

To verify that the functional cluster of transcriptome sequences analysis was reliable, 11 *PbrLBD* genes were selected to conduct a qRT-PCR experiment to investigate the expression levels in six different tissues of the ‘Dangshansuli’ pear, including stem, leaf, petal, fruit, sepal and ovary (Fig. 6). Results showed that expression of all 11 *PbrLBD* genes was detected in the six tissue types. Moreover, the 11 *PbrLBD* genes exhibited a diversity of expression patterns in the six different tissues, suggesting that *PbrLBD* genes may function in different tissues and participate in diverse metabolic processes. Previous studies had reported that *LBD* genes strongly expressed in the micropylar side of the mature ovary [26]. In our study, seven genes (*PbrLBD54*, *PbrLBD57*, *PbrLBD33*, *PbrLBD30*, *PbrLBD53*, *PbrLBD43* and *PbrLBD50*) exhibited a similar expression pattern with a high expression level in ovary tissues, suggesting that *PbrLBD* genes play critical functions during ovary development. The *PbrLBD20* and *PbrLBD25* genes were highly expressed in stem tissues, and *PbrLBD10* genes were highly expressed in fruit. Overall, these *PbrLBD* genes may play critical roles in pear growth and development processes.

#### Potential functions of *PbrLBD* genes in anthocyanin biosynthetic pathway

**Fig. 7:**
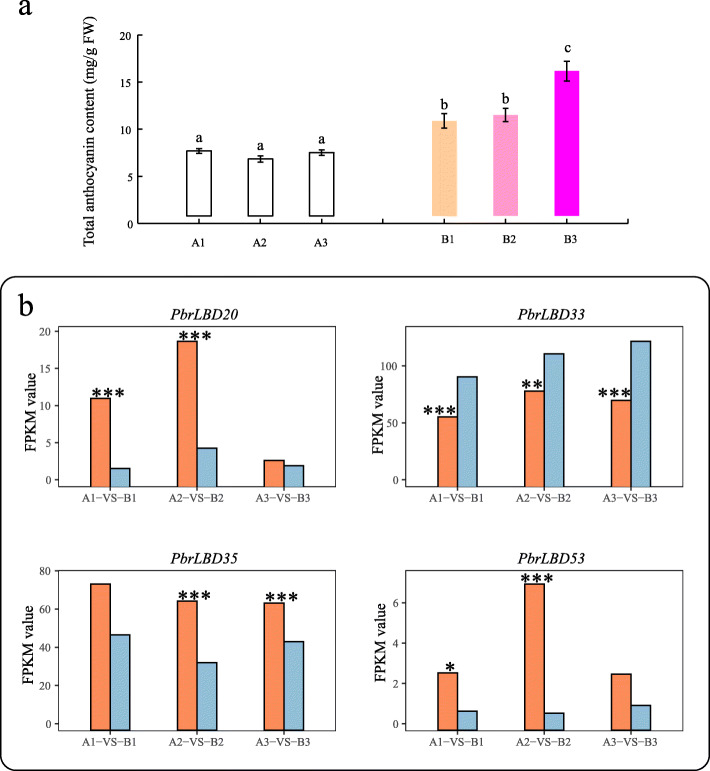
The candidate *PbrLBD* gene associated with anthocyanin biosynthetic pathway. (**a**) Dynamic changes of total anthocyanin content (mg/g FW) in peels at different library. B1, B2 and B3 represent the fruits sampled at 4, 8 and 10 days after bag removal, and three bagged fruits (A1, A2, A3) also were sampled at the same time, as a control (Duncan test, *P* < 0.05). (**b**) The FPKM (Fragments Per Kilobase per Million) values of four *PbrLBD* genes (*PbrLBD20*, *PbrLBD33*, *PbrLBD35*, *PbrLBD53*) related to anthocyanin biosynthetic pathway at three comparison, including A1-VS-B1, A2-VS-B2, A3-VS-B3. The x-axis represents the three different comparisons, and the y- axis represents the FPKM values. Note: A-VS-B, orange bar represents A, light-blue bar represents B (*P < 0.05, **P < 0.01 and ***P < 0.001, *T-test*)

Previous studies have revealed that some *LBD* genes can act as important transcription factors in regulating anthocyanin accumulation [2, 27]. To validate the potential functions of *PbrLBDs* in the anthocyanin biosynthetic pathway, we further analyzed our previous pear transcriptome data [28]. Compared to bagged fruits, the fruits removed from bags had significant higher anthocyanin concentrations in three comparisons (Fig. 7a). Differentially expressed genes (DEGs) analysis results indicated that four genes (*PbrLBD20*, *PbrLBD33*, *PbrLBD35* and *PbrLBD53*) were differentially expressed in two or more comparisons of bagged fruit and fruit removed from bags (Fig. 7b). As shown in Fig. 7b, *PbrLBD33* genes were found to be significantly up-regulated in pears with high anthocyanin concentrations in all three comparisons. Furthermore, MYB binding sites involved in flavonoid biosynthetic gene regulation were found in the promotors of the *PbrLBD33* gene. These results suggested that *PbrLBD33* may participate positively in anthocyanin biosynthesis. However, expression of the other four DEGs was found to be down-regulated in pears with high anthocyanin concentrations, suggesting that these genes may repress anthocyanin accumulation. These results provided strong evidence that these *LBD* genes act as critical regulators in the anthocyanin biosynthetic pathway.

### Subcellular localization of PbrLBD20 protein

**Fig. 8:**
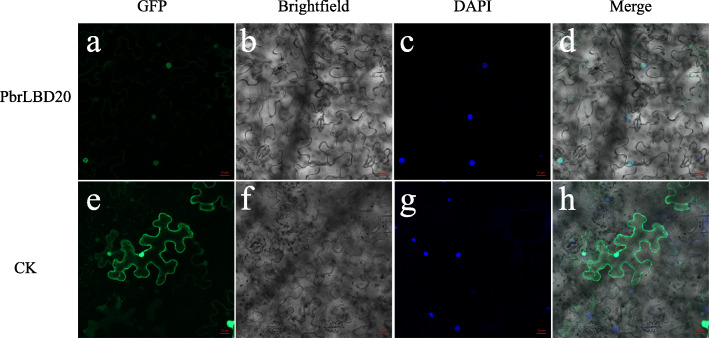
Subcellular localization of PbrLBD20. PbrLBD20 was nuclear localized as determined by 35S:PbrLBD20–GFP fusion protein in tobacco leaf epidermal cells. Transient expression of GFP alone (35S:GFP) in tobacco leaf epidermal cells was used as a control (CK). The nucleus was identified by DAPI staining were used as markers. Images were taken in a dark field for green fluorescence, while the outline of the cells and the merged image were recorded in a bright field. Scale bars =20 μm

In our study, the *LBD* gene was a type of plant-specific transcription factor. *PbrLBD20* was selected from four candidate anthocyanin-related genes for further subcellular localization, and its protein was predicted to be located in the nucleus. To observe the subcellular localization of *PbrLBD20*, a plasmid with the GFP gene fused to PbrLBD20 CDS and driven by the 35S promoter was translocated into tobacco leaves. A confocal microscope was used to determine the localization of recombinant *PbrLBD20*. In Fig. 8, we observe that CK (empty vector) is located at the membrane and the nucleus, and PbrLBD20-GFP shows strong signals in the nucleus (Fig. 8). This result validates that *PbrLBD20* is a transcription factor.

## Discussion

### Whole-genome identification and phylogenetic analysis of *LBD* genes in pear

As plant-specific transcription factors, *LBD* genes encode a conserved LOB (lateral organ boundaries) domain and are functional in diverse biological processes, including abiotic stress responses, anthocyanin biosynthesis, nitrogen metabolism and lateral organ development. Given their important function during plant development, *LBD* genes have been identified in different plant species, such as apple (58) [11], rice (35), maize (44) [12], *Arabidopsis thaliana* (42) [5] and grape (40) [13]. Pear is one of the most important fruit trees, however, research on *LBD* genes in pear has been relatively rare. In our study, a relatively strict criterion was performed to determine candidate members of the *LBD* gene family in pear. The candidate genes lacked a conserved LOB domain, and genes that were unanchored on a chromosome were removed from consideration. With these criteria, a total of 60 *PbrLBD* genes were identified, which is similar to the number identified for apple (58) [11]. According to the presence/absence of a conserved LX6LX3LX6L leucine zipper-like domain in the C terminus, members of the *LBD* gene family were divided into two classes (Fig. 1). Among them, 53 *PbrLBD* genes were classified as class I and six were classified as class II (Additional file 7: Figure S4). Previous studies had reported that 84% of members of the LBD gene family that were found in *Arabidopsis* and 78% of those found in apple belonged to class I [8, 11]. This result provided further evidence that a higher percentage of *LBD* genes found in different species fall into class I versus class II.

Based on the phylogenetic grouping relationships, a total of 159 *LBD* genes in three different species were organized into seven major subgroups (Ia, Ib, Ic, Id, Ie, IIa and IIb). Although there are differences, this phylogenetic tree was largely consistent with the phylogenetic tree results from a previous study [8, 11]. Homologous genes with similar functions typically will be clustered into the same subclades, which provides a valuable reference to predict gene function [12] (Fig. 1). Previous studies have reported that class II genes may play crucial roles in anthocyanin biosynthesis [12]. For example, one study found that *ASL39/LBD37*, *ASL40/LBD38* and *ASL41/LBD39* could negatively regulate anthocyanin biosynthesis by responding to Nitrogen (N) and nitrate (NO3^−^) [2]. Therefore, we speculated that class II *LBD* genes of pear might play important roles in anthocyanin biosynthesis.

### The evolution history of *PbrLBD* gene family

Previous studies have reported that the expansion of gene families were mainly driven by gene duplication, such as WGD event/segmental duplication, and dispersed and tandem duplication [29, 30]. For example, the expansion of *AP2/ERF* and *WRKY* gene families were primarily driven by WGD/segmental duplication and tandem duplications [31, 32]. The *NBS-LRR* gene family is thought to have expanded by transposed duplications [33]. There are at least two WGD/segmental duplication events that occurred in the evolution process of the pear genome, including an ancient WGD event (approximately ~ 140 MYA) and a relatively recent WGD event (approximately 30–45 MYA) [19, 34]. *Synteny* analysis indicated that WGD event/segmental duplication played a leading role in the expansion of the *PbrLBD* gene family. However, our results of mean Ks and 4DTv values of *PbrLBD* paralogs showed that the *PbrLBD* gene family had undergone only one recent WGD/segmental duplication event (30–45 MYA) [20]. It is interesting to note that five duplicated gene pairs (*PbrBD60*/*PbrBD59*, *PbrBD14*/*PbrBD42*, *PbrBD26*/*PbrBD6*, *PbrBD57*/*PbrBD33* and *PbrBD13*/*PbrBD41*) exhibited relatively low mean Ks values, ranging from 0.008 to 0.14, suggesting that a more recent gene duplication event occurred in the evolution process of the *PbrLBD* gene family. Following duplication, duplicated gene pairs can undergo three different selection pressures, including positive selection, negative selection (purifying selection) and neutral selection [35]. Our analysis indicated that the evolution of the *PbrLBD* gene family was mainly driven by purifying selection.

### The potential roles of Chinese white pear *LBD* transcription factors

Increasing evidence indicates that *LBD* genes play diverse roles during plant development processes. In general, the gene expression patterns can provide important clues to predict the gene function [36]. Transcriptome sequencing analysis and qRT-PCR expression profiling were conducted to investigate *LBD* gene expression patterns in six different tissues. The members of the LBD gene family exhibited a variety of expression patterns in six different tissues, even in a single subclass. This result indicated that *PbrLBD* genes may participate in various biological processes. Ten genes were expressed in all tissues, suggesting these genes play a broad role in pear growth and development. Additionally, 73.33% of *PbrLBDs* were detected in transcriptional abundance in pear sepal, suggesting these genes may have important roles in the development of pear sepal. Further, the expression levels of 11 randomly selected members of the *PbrLBD* gene family were validated using qRT-PCR. Based on qRT-PCR expression profiles, we found that most of the 11 *PbrLBD* genes were highly expressed in flower organ, such as petal, sepal and ovary. Therefore, we predicted that *LBD* genes may function in reproductive organs. A previous study reported that *AtASL1* genes were involved in flower development through controlling the cell fate determination in *Arabidopsis* petals [37]. As shown in Fig. 1a, *PbrLBD10* and *PbrLBD53* were grouped into the same subclades with *AtASL1*, and exhibited relatively high expression levels in pear petals. This result indicated that *PbrLBD10* and *PbrLBD53* play an important role in petal development. Interestingly, we found that *PbrLBD10* was highly expressed in mature fruit. In grape, the expression levels of the two *LBD* genes (*GSVIVT01008284001* and *GSVIVT01024592001*) were low expressed before the fruit color change period, while were gradually increased during fruit ripening [38]. In banana, the expression level of *MaLBD1*, *MaLBD2* and *MdLBD3* were increased during fruit ripening, and these three genes involved in regulating fruit ripening by activating the transcription of cell wall-loosening-related genes *MaEXPs* [39]. Similarly, we speculated that *PbrLBD10* gene might involve in the regulation of fruit ripening and related color changes. However, the function of *LBD* genes in fruit is still unknown and needs to be investigated further in future studies.

Anthocyanins are important flavonoid compounds detected in plants that are beneficial to human health [40, 41]. In pear, anthocyanin concentration determines fruit quality and color. However, anthocyanin accumulation is influenced by many environmental conditions, such as temperature, light and drought [42]. Numerous studies have indicated that *LBD* genes play important roles in anthocyanin biosynthesis processes. In apple, the overexpression of *MdLBD13* negatively regulated anthocyanin accumulation through repressing the expression levels of structural genes in the flavonoid pathway [27]. In *Arabidopsis thaliana*, *ASL39/LBD37*, *ASL40/LBD38* and *ASL41/LBD39* negatively regulated anthocyanin biosynthesis in response to N/NO_3_^−^ [8]. In this study, transcription sequencing data from pear fruits after bagging and bag removal indicated that *PbrLBD20*, *PbrLBD33*, *PbrLBD35* and *PbrLBD53* may act as important regulators in anthocyanin accumulation*. Interestingly, the light-responsive elements were found in the promoters for all four of these genes. This result indicated that these four PbrLBD genes may be involved in* light*-responsive* anthocyanin *biosynthesis.*

## Conclusions

In our study, a total of 60 *LBD* genes were identified in pear genome, with an unevenly distributed among chromosomes. According to gene structure and phylogenetic analysis, the *PbrLBD* gene family was divided into two classes: class I (53) and class II (7). Synteny analysis further indicated that WGD/segmental duplication (30–45 MYA) played a key role in the expansion of *PbrLBD* gene family. Purifying selection was primary force during *PbrLBD* gene family evolution process. Based on mean Ks values and 4DTV values, *PbrLBD* gene family undergone only one recent WGD event occurring at 30–45 MYA. Transcription sequencing data from pear fruits after bagging and bag removal further indicated that *PbrLBD33* may positively regulate the anthocyanin accumulation, while genes *PbrLBD20*, *PbrLBD35* and *PbrLBD53* act as negative roles. Interestingly, the light-responsive elements were found in the promoters for all four of these genes. This result indicated that these four *PbrLBD* genes may be involved in light-responsive anthocyanin biosynthesis. Further functional analysis indicated *PbrLBD20* was localized in the nucleus. These results provide valuable information for understanding the evolution of the *PbrLBD* gene family, and facilitate further research on functional characterization of *PbrLBD* genes in anthocyanin biosynthesis in future studies.

## Methods

### Whole-genome identification of *LBD* gene family in Chinese white pear

The pear (*Pyrus bretschneideri*) genome sequence was downloaded from the pear genome project (http://peargenome.njau.edu.cn/) [20] and the *Arabidopsis* LBD protein sequences were downloaded from the Plant Transcription Factor Database (PlantTFDB) (http://planttfdb.cbi.pku.edu.cn). First, all pear protein sequences were scanned by BLASTP using all *Arabidopsis* LBD protein sequences as queries [12]. Second, we performed a hidden Markov model search (HMMsearch) for the pear protein database using the HMM profile with the LOB domain (PF00847) [8]. The redundant sequences between the results of HMMsearch and BLASTP were removed. Then, the CDD tool (https://www.ncbi.nlm.nih.gov/cdd), SMART tool (http://smart.emblheidelberg.de/) and InterProScan tool (http:// www.ebi.ac.uk/Tools/pfa/iprscan/) were used to verify the completeness of the LOB domain. The distribution of identified *LBD* genes across chromosomes were extracted from the pear genome database using an in-house Python script, and displayed in Figure S1 by using MapChart [43]. In additional, we predicted the protein molecular weights and isoelectric points of pear *LBD* genes on the ExPASy proteomics server, an online proteomics and sequence analysis tool (http://expasy.org) [44].

### Gene structure and conserved motif analysis

Gene structural information for *PbrLBD* genes was obtained from the pear genome database and displayed using TBtools software [45]. The MEME tool (http://meme-suite.org/tools/meme) was used to identify the conserved domain motif by multiple alignment analysis [46]. The parameters were set as following: repetitions, any number; number of different motifs, 20; minimum motif width, 30; maximum motif width, 70. ClustalW analysis was performed using the full-length protein sequences of *PbrLBD* genes with default multiple alignment parameters and visualization was achieved with GeneDoc software [47, 48].

### Multiple sequence alignment and phylogenetic analysis

The construction of the phylogenetic tree followed a two-step process. First, all LBD full-length protein sequences in *Pyrus bretschneideri*, *Arabidopsis thaliana* and *Malus domestica* were aligned using ClustalW with default parameters [47]. Then, an unrooted phylogenetic tree was constructed by MEGA-X based on the result of multiple sequence alignment using the NJ (Neighbor-Join) method with 1000 bootstrap replicates [49]. Evolview (version 2) [50], an online visualization tool, was used to visualize the phylogenetic tree.

### Cis-regulatory elements analysis of *PbrLBD* gene promoters

Bedtools software was used to extract the 2000-bp upstream (putative promoter region) sequences of the transcription start site of all *PbrLBD* genes [51]. Then, PlantCare was used to predict the cis-acting elements in the putative promoter region of *PbrLBD* genes [18].

### Synteny analysis of *PbrLBD* genes

We performed synteny analysis to identify synteny blocks using a method described in the PGDD database (http://chibba.agtec.uga.edu/duplication/). First, a BLASTP alignment was conducted to search for candidate homologous gene pairs across the whole genome of Chinese White pear, and the parameters were set to: E-value, 1e-10; −max_target_seqs, 5 [52]. To identify syntenic chains, these candidate homologous gene pairs served as the input for MCScanX software (default parameters) [53]. DupGen_finder-unique was further used to distinguish the whole-genome duplication (WGD) /segmental duplication, single duplication (SD), tandem duplication (TD), proximal duplication (PD), and transposed duplication (TRD), with the priority of the duplication genes as follows: WGD > TD > PD > TRD > DD [54]. The synteny and position information of *LBD* genes was displayed by plotting a graph using Circos software (version 0.69.2).

### Calculating Ka, Ks and 4DTv of *PbrLBD* paralogs

ParaAT 2.0 [55] and Kaks_calculator 2.0 [56] were used to calculate the Ka, Ks values of *PbrLBD paralog* gene pairs located in the same synteny block. The Kaks_calculator 2.0 parameters were set as follows: YN as the Method (−m) and Standard Code as the Genetic code table (−c). 4DTv (4-fold synonymous third-codon transversion) was widely used to estimate the genetic distances of synteny gene pairs. We calculated 4DTv values of *PbrLBD paralogs using our in-house Python script.* The distribution of mean Ks and 4DTv values of 22 paralogs were plotted using the hist function in R.

### Expression analysis by ESTs

A local BLASTN alignment against Chinese white pear EST libraries was conducted to find the corresponding records for each member of the *PbrLBD* gene family using the following parameters: maximum identity > 95%, length > 200 bp, and E-value < 10^− 10^.

#### Expression pattern analysis of *PbrLBD* genes by transcriptome sequencing

The RNA-seq data in six different tissues of the cultivar ‘Dangshansuli’ were acquired from our previous study, including stem, leaf, sepal, petal, fruit and ovary [57]. The RNA-seq data from sand pear cultivar ‘Mantianhong’ (*Pyrus pyrifolia*) samples comparing bagging and removal from bags also was acquired from our previous study [28]. We bagged the fruits at 35 days after full bloom (DAFB), and then bag removal at 10 days before commercial maturity. B1, B2 and B3 represent the fruits sampled at 4, 8 and 10 days after bag removal, and three bagged fruits (A1, A2, A3) also were sampled at the same time, as a control. We downloaded them from the National Center for Biotechnology Information database (NCBI, https://www.ncbi.nlm.nih.gov/). Heatmap.2, a function in R, was used to plot heatmaps based on the log2(RPKM+ 1) value of each *PbrLBD* gene.

##### The measurement of anthocyanins content

Anthocyanins were extracted and measured according to the method described by previous study [58].

### Quantitative real-time PCR analysis (qRT-PCR)

Samples of the six different tissues in “Dangshansuli” including stem, leaf, petal, fruit, sepal and ovary were collected for qRT-PCR analysis. Total RNA was extracted from mixed samples of the six different tissues using a Plant Total RNA Isolation Kit Plus (FOREGENE Co. Ltd., Chengdu, China). Then, total RNA concentrations were adjusted to all be the same, and then first-strand cDNA was synthesized using TransScript One-Step gDNA Removal and cDNA Synthesis SuperMix (TransGen Biotech Co. Ltd., Beijing, China). Eleven pairs of the most specific primers were designed by using NCBI online software (https://www.ncbi.nlm.nih.gov/tools/primerblast), which amplified the 11 candidate gene sequences (Additional file 8: Table S4). qRT-PCR analysis was performed using LightCycler 480 SYBR GREEN I Master (Roche). A 20-μl mixed reaction system was set up. Each sample contained 150 ng of template cDNA, 0.5 μM of each primer pair and 10 μl of LightCycler 480 SYBR GREEN I Master. All reactions were set up in 96-well plates and each cDNA sample had three replicates. We set the qRT-PCR conditions as follows: first 5 min at 95 °C for pre-incubation, 55 cycles at 95 °C for 3 s, 60 °C for 10 s, and 72 °C for 30 s, and then 3 min at 72 °C for extension. Finally, fluorescence signal data collection was carried out at 60 °C. Pyrus TUB (No. AB239681) was used for internal control genes (Additional file 8: Table S4). The average threshold cycle (Ct) for each cDNA sample was calculated using the running results displayed on the computer. At the same time, the relative expression levels of the seven genes were calculated using the 2^-ΔΔCt^ method, which is described in the previous study [59].

#### Subcellular localization analysis

For subcellular localization analysis of *PbrLBD20*, a 35S-PbrLBD20-GFP fusion vector was constructed according to Xue et al. CE Design v1.04 (Vaezyme, China) was used to design specific primers for cloning and recombination [60]. Agrobacterium transformation (*Agrobacterium tumefaciens* strain GV3101) was conducted using the freeze-thaw method after the fusion vector was confirmed by sequencing. The empty GFP vector was used as a control. Then, the agrobacterium suspensions (OD_600_ = 0.8 ~ 1.0) were injected into the abaxial surfaces of 3-week-old *Nicotiana benthamiana* plant leaves. After three days of culturing in a greenhouse, the injection sites and their surrounding tissues were collected for vacuum treatment and immediately stained with DAPI. Then, a Leica TCs SP2 spectral confocal microscope (Leica Microsystems, Germany) was used to observe green fluorescence and nuclear DNA signals. ZEN 2012 (Carl Zeiss Microscopy GmbH, Germany) and Adobe Illustrator 2020 (Adobe Systems, USA) were used for image processing.

#### Statistical analysis

Anthocyanin content were analyzed with SPSS 20 software (IBM, USA). One-way ANOVA followed by Duncan test was used to compare the statistical difference at the significance level of *P* < 0.05. The *T-test* were conducted to evaluate the significant differences of gene expression level between bagged and bag removal treatment (*P < 0.05, **P < 0.01 and ***P < 0.001).

## Supplementary information

**Additional file 1: Figure S1**. Conserved domains of PbrLBD protein family. (a) Multiple sequence alignment of PbrLBD proteins by ClustalW. The result of multiple sequence alignment was visualized by GeneDoc tool. (b) The logos of the CX2CX6CX3C zinc finger-like domain and the LX6LX3LX6L leucine zipper-like domain.

**Additional file 2: Table S1.** The members of *PbrLBD* gene family in Chinese White Pear genome.

**Additional file 3: Figure S2.** The location of *PbrLBD* genes on pear chromosomes.

**Additional file 4: Figure S3.** The number of *LBD* genes in five duplication types.

**Additional file 5: Table S2.** The synteny block contained *PbrLBD* duplicated gene pairs.

**Additional file 6: Table S3.** The synteny analysis of *PbrLBD* gene family.

**Additional file 7: Figure S4**. The percentage of *LBD* genes of each class in three species, including apple, pear and *Arabidopsis*.

**Additional file 8: Table S4.** The eleven pairs of primer sequences for amplifying *PbrLBD* genes by qRT-PCR analysis.

## Data Availability

The pear (*Pyrus bretschneideri*) genome sequence and RNA-seq data was downloaded from the pear genome project (http://peargenome.njau.edu.cn/).

## Abbreviations

LBD: Lateral organ boundaries domain
TF: Transcription factor
pI: Isoelectric point
WGD: Whole genome duplication
DD: Dispersed duplications
TD: Tandem duplication
PD: Proximal duplication
TRD: Transposed duplication
GA: Gibberellin
ABA: Abscisic acid
MeJA: Methyl Jasmonate
MYB: Myeloblastosis
4DTv: 4-fold synonymous (degenerative) third-codon transversion
MYA: Million years ago
qRT-PCR: Quantitative real time polymerase chain reaction
DEGs: Differentially expressed genes
