# Supplementary material for: Mining and evolution analysis of lateral organ boundaries domain (LBD) genes in Chinese white pear (Pyrus bretschneideri)
Source: BMC Genomics. 2020 Sep 21;21:644. doi: 10.1186/s12864-020-06999-9 (PMC7504654; doi:10.1186/s12864-020-06999-9)
Supplement: Supplementary file 1 — Additional file 1: Figure S1. Conserved domains of PbrLBD protein family. (a) Multiple sequence alignment of PbrLBD proteins by ClustalW. The result of multiple sequence alignment was visualized by GeneDoc tool. (b) The logos of the CX2CX6CX3C zinc finger-like domain and the LX6LX3LX6L leucine zipper-like domain. [file 12864_2020_6999_MOESM1_ESM.docx]

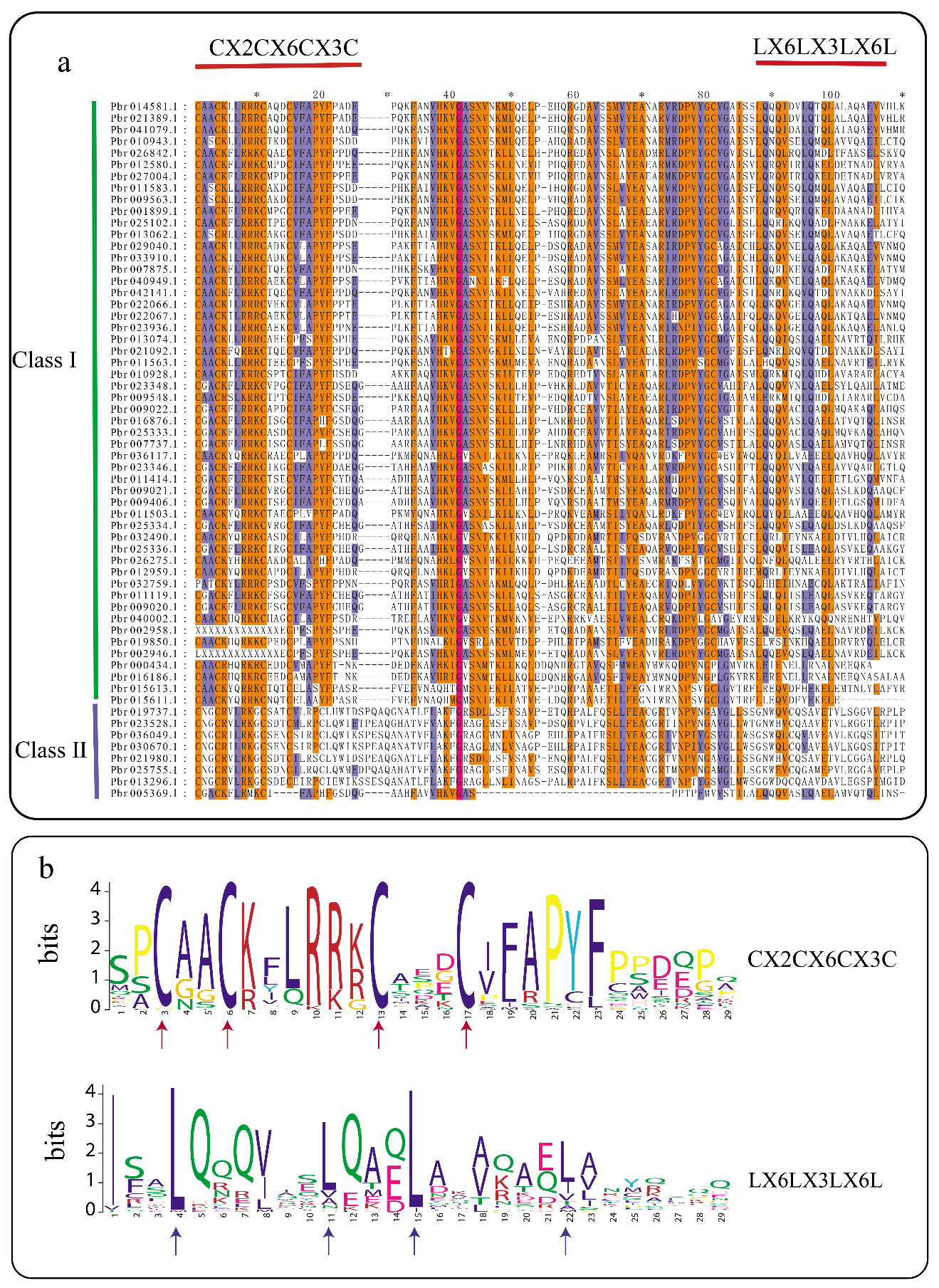


**Figure S1.** Multiple sequence alignment and conserved domains of PbrLBD protein family. (a) The amino acid sequences of PbrLBD proteins were aligned by ClustalW in Mega software. The result of multiple sequence alignment was visualized by GeneDoc tool. (b) The logos of the CX2CX6CX3C zinc finger-like domain and the LX6LX3LX6L leucine zipper-like domain. Conserved domains were predicted by MEME (Multiple Em for Motif Elicitation) tool using the amino acid sequences of PbrLBD proteins.
