# Supplementary figures and images for: Mining and evolution analysis of lateral organ boundaries domain (LBD) genes in Chinese white pear (Pyrus bretschneideri)
Source: BMC Genomics. 2020 Sep 21;21:644. doi: 10.1186/s12864-020-06999-9 (PMC7504654; doi:10.1186/s12864-020-06999-9)

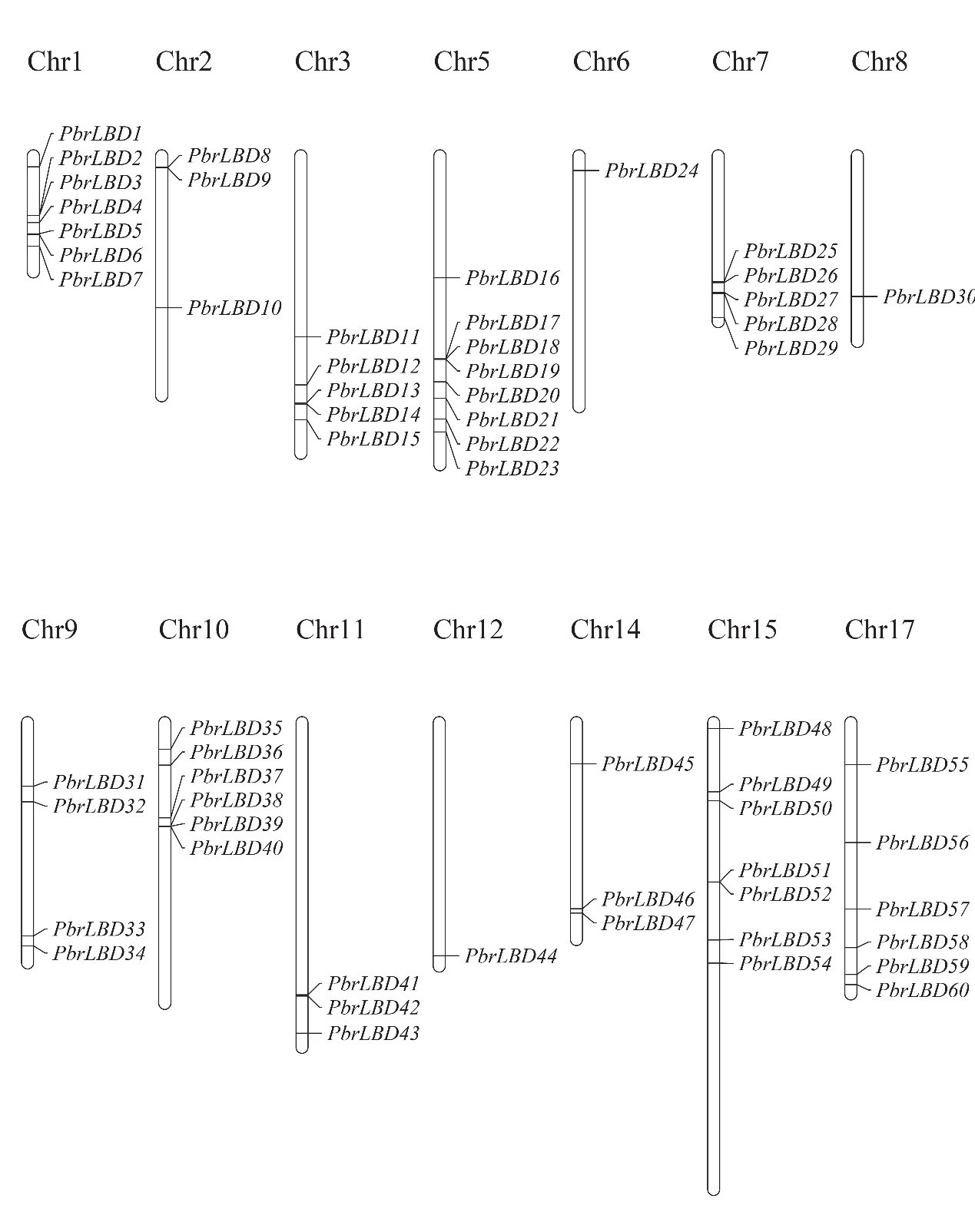


**Figure S2.** The location of *PbrLBD* genes on 17 pear chromosomes.

Supplement: Supplementary file 3 — Additional file 3: Figure S2. The location of PbrLBD genes on pear chromosomes. [file 12864_2020_6999_MOESM3_ESM.docx]
