# Supplementary material for: Mining and evolution analysis of lateral organ boundaries domain (LBD) genes in Chinese white pear (Pyrus bretschneideri)
Source: BMC Genomics. 2020 Sep 21;21:644. doi: 10.1186/s12864-020-06999-9 (PMC7504654; doi:10.1186/s12864-020-06999-9)
Supplement: Supplementary file 4 — Additional file 4: Figure S3. The number of LBD genes in five duplication types. [file 12864_2020_6999_MOESM4_ESM.docx]

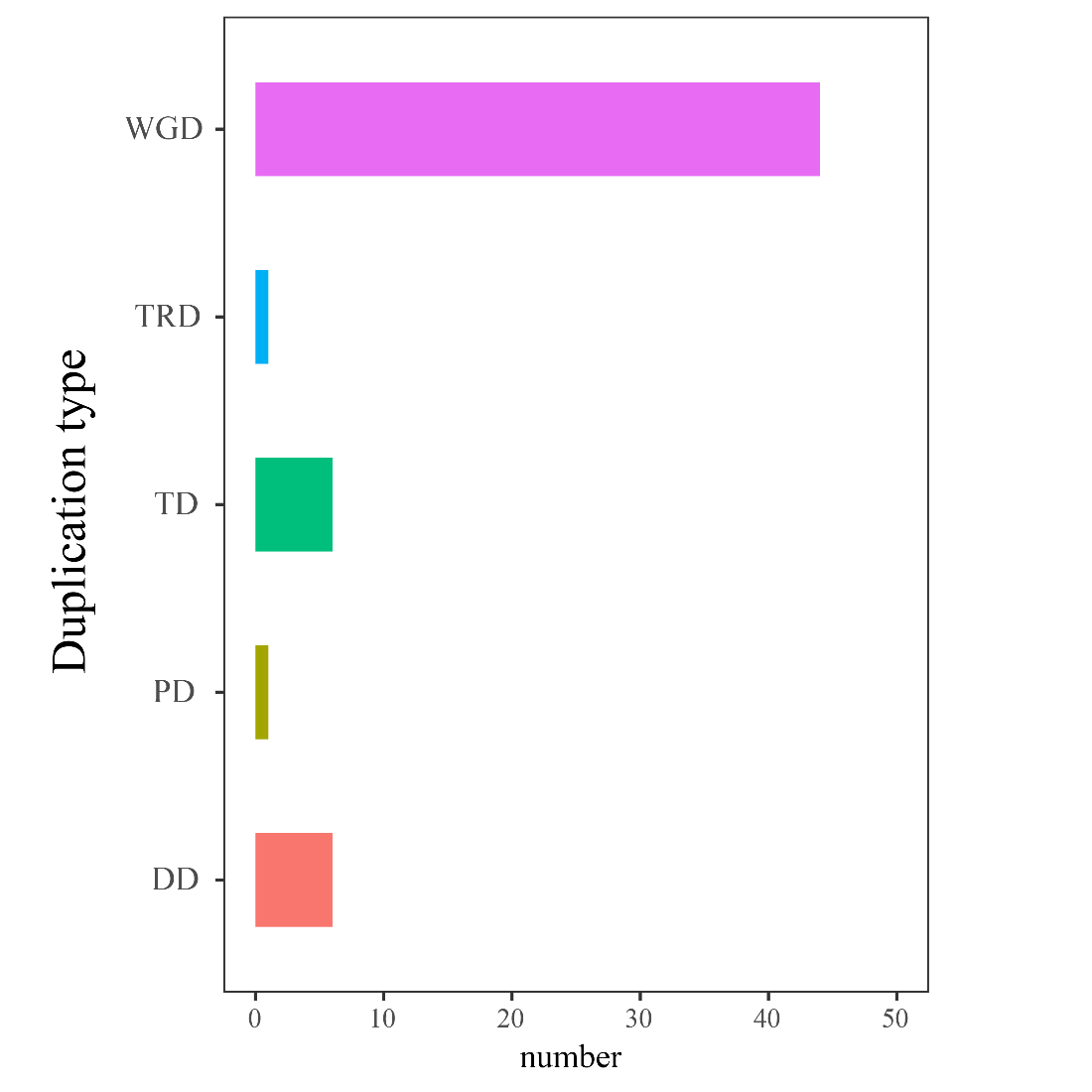


**Figure S3**. The number of *PbrLBD* genes duplicated from five duplication events. The x-axis represents the number of *PbrLBD* gene in five gene duplication events, and the y- axis represents five gene duplication events. WGD indicates whole-genome duplication/segmental duplication; DD indicates dispersed duplication; TD indicates tandem duplication; PD indicates proximal duplication; and TRD indicates transposed duplication.
