# Supplementary material for: Mining and evolution analysis of lateral organ boundaries domain (LBD) genes in Chinese white pear (Pyrus bretschneideri)
Source: BMC Genomics. 2020 Sep 21;21:644. doi: 10.1186/s12864-020-06999-9 (PMC7504654; doi:10.1186/s12864-020-06999-9)
Supplement: Supplementary file 7 — Additional file 7: Figure S4. The percentage of LBD genes of each class in three species, including apple, pear and Arabidopsis. [file 12864_2020_6999_MOESM7_ESM.docx]

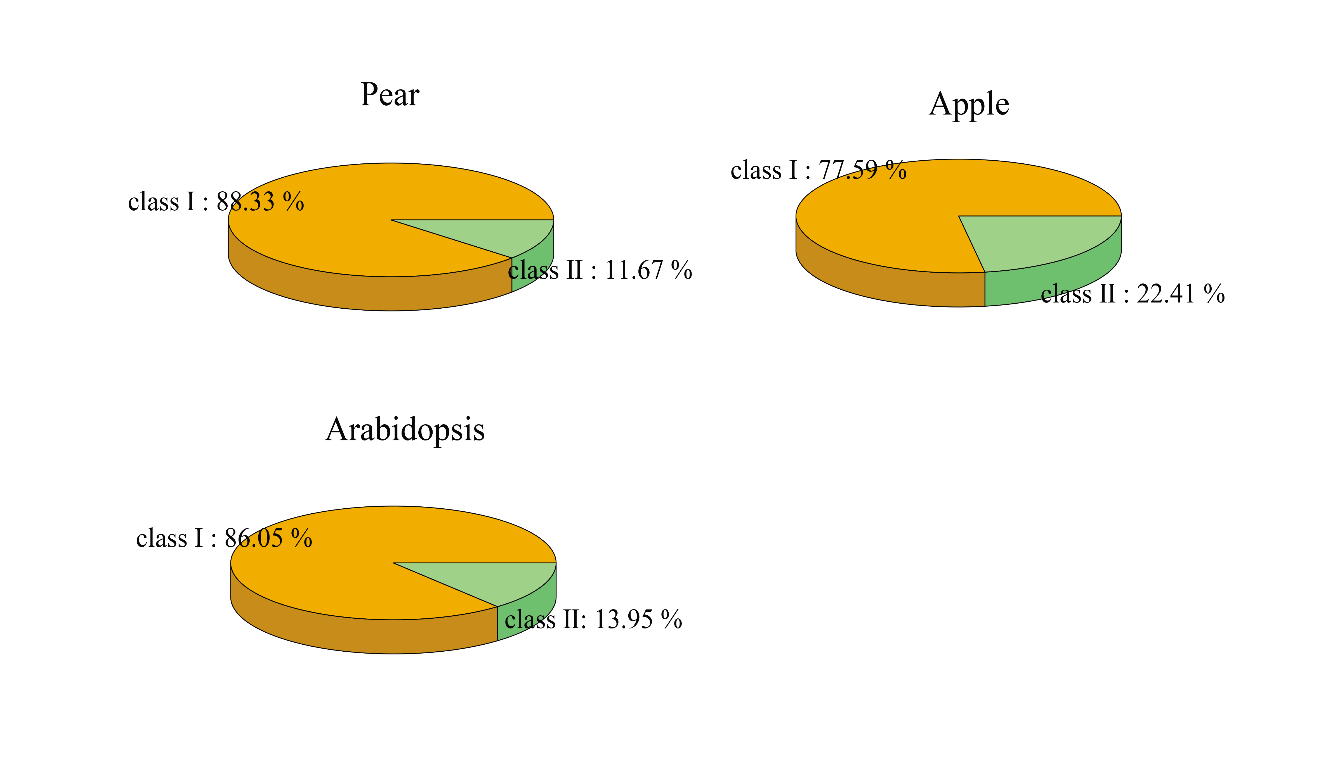


**Figure S4**. The percentage of *LBD* genes of each class in three species, including apple, pear and *Arabidopsis*. The orange part indicates the proportion of *LBD* gene in class Ⅰ, the green part indicates the proportion of *LBD* gene in class Ⅱ.
